# The case of the youngest infant with hepatoblastoma with APC gene mutation

**DOI:** 10.1186/1897-4287-10-S3-A18

**Published:** 2012-04-20

**Authors:** Maryna Krawczuk-Rybak, Anna Jakubiuk-Tomaszuk, Elżbieta Skiba, Andrzej Pławski

**Affiliations:** 1 Department of Pediatric Oncology and Haematology, Medical University of Bialystok, Poland; 2 Department of Pediatric Laboratory Diagnostics, Medical University of Bialystok, Poland; 3 Institute of Human Genetics, Polish Academy of Sciences, Poznan, Poland

## 

Hepatoblastoma is a rare early malignant liver neoplasm occurring in infants and children. Some cases of hepatoblastoma are associated with genetic conditions such as trisomies of chromosomes 18, Beckwith-Wiedemann syndrome and familial adenomatous polyposis (FAP). The observed increase in the risk of hepatoblastoma in APC (adenomatous polyposis coli) gene mutation carriers is low, not exceeding 1%, which is associated with APC gene mutation located in the region of codons 459-1309, where most mutations identified in families affected by FAP in Poland are found. Tissues obtained from patients with hepatoblastoma but without the diagnosis of FAP show loss of heterozygocity at the locus of APC or MCC (mutated in colorectal cancers) genes. Hepatoblastoma growth is the result of the sequence of changes in genetic material. However, a major role can be ascribed to the Wnt pathway where somatic mutations have been observed in the genes of Bcathenin (CTNNB1, Cathenin (cadherin-associated protein), beta 1.88kD) and AXIN1 (Axis inhibitor 1). We present a case of familial hepatoblastoma in a 3-month-old infant with a constitutional mutation in the APC gene.

